# Carbon ion radiotherapy boost in the treatment of glioblastoma: a randomized phase I/III clinical trial

**DOI:** 10.1186/s40880-019-0351-2

**Published:** 2019-02-20

**Authors:** Lin Kong, Jing Gao, Jiyi Hu, Rong Lu, Jing Yang, Xianxin Qiu, Weixu Hu, Jiade J. Lu

**Affiliations:** 1 Department of Radiation Oncology, Shanghai Proton and Heavy Ion Center, Fudan University Shanghai Cancer Center, Shanghai, 201321 P. R. China; 2 Department of Radiation Oncology, Shanghai Proton and Heavy Ion Center, Pudong, 4365 Kangxin Road, Shanghai, 201321 P. R. China

**Keywords:** Glioblastoma, Anaplastic astrocytoma, Carbon ion radiotherapy, Proton radiotherapy, Temozolomide, Overall survival, Progression-free survival, Toxicity, Serologic immune response, O-6-methylguanine-DNA methyltransferase

## Abstract

**Background:**

Glioblastoma (GBM) is a highly virulent tumor of the central nervous system, with a median survival < 15 months. Clearly, an improvement in treatment outcomes is needed. However, the emergence of these malignancies within the delicate brain parenchyma and their infiltrative growth pattern severely limit the use of aggressive local therapies. The particle therapy represents a new promising therapeutic approach to circumvent these prohibitive conditions with improved treatment efficacy.

**Methods and design:**

Patients with newly diagnosed malignant gliomas will have their tumor tissue samples submitted for the analysis of the status of O-6-methylguanine-DNA methyltransferase (*MGMT*) promoter methylation. In Phase I, the patients will undergo an induction carbon ion radiotherapy (CIRT) boost followed by 60 GyE of proton irradiation with concurrent temozolomide (TMZ) at 75 mg/m^2^. To determine the maximal dose of safe induction boost, the tolerance, and acute toxicity rates in a dose-escalation manner from 9 to 18 GyE in three fractions will be used. In Phase III, GBM-only patients will be randomized to receive either 60 GyE (2 GyE per fraction) of proton irradiation with concurrent TMZ (control arm) or a CIRT boost (dose determined in Phase I of this trial) followed by 60 GyE of proton irradiation with concurrent TMZ. The primary endpoints are overall survival (OS) and toxicity rates (acute and long-term). Secondary endpoints are progression-free survival (PFS), and tumor response (based upon assessment with C-methionine/fluoro-ethyl-tyrosine positron emission tomography [MET/FET PET] or magnetic resonance imaging [MRI] and detection of serologic immune markers). We hypothesize that the induction CIRT boost will result in a greater initial tumor-killing ability and prime the tumor microenvironment for enhanced immunologic tumor clearance, resulting in an expected 33% improvement in OS rates.

**Discussion:**

The prognosis of GBM remains grim. The mechanism underpinning the poor prognosis of this malignancy is its chronic state of tumor hypoxia, which promotes both immunosuppression/immunologic evasion and radio-resistance. The unique physical and biological properties of CIRT are expected to overcome these microenvironmental limitations to confer an improved tumor-killing ability and anti-tumor immune response, which could result in an improvement in OS with minimal toxicity.

*Trial registration number* This trial has been registered with the China Clinical Trials Registry, and was allocated the number ChiCTR-OID-17013702.

## Background

Glioblastoma (GBM) is the most biologically aggressive and the most commonly diagnosed primary tumor of the brain. It is characterized by a rapid progression and diffuse infiltration of the brain parenchyma. While a modest improvement in overall survival (OS) was achieved with the addition of temozolomide (TMZ), which has now become the standard of care, the expected median survival remains dismal at around 15 months [1]. Attempts at intensifying the TMZ dosing has proved to be ineffective [2]. Numerous lines of investigation on alternative treatments, both historical and ongoing, have included boron capture therapy, altered radiotherapy fractionation, polychemotherapy regimens, vaccination, and targeted biological agents, but all have failed to become the standard of care. Clearly, alternative approaches are needed to improve the outcomes of these patients.

### GBM-tumor hypoxia and immunology

A hallmark characteristic of GBM is the presence of tumor cell hypoxia. From a cancer biology and tumor evolution perspective, hypoxia is a physiologic stressor that promotes the selection of a more aggressive phenotype exhibited by aberrant cell populations. Severe hypoxia is known to induce an increased invasiveness, dysregulated angiogenesis, increased mutagenicity, altered metabolism, and increased proliferation of GBM tumor cells [3–5]. From an anti-tumor perspective, hypoxia significantly limits the effectiveness of TMZ chemotherapy, and that of photon-based radiotherapy, which relies on oxygen-derived free-radical species to confer an indirect DNA damage towards killing tumor cells. This enhances the expression of stem cell markers, as stem-like properties impart great cellular resistance to all types of cytotoxic therapy [6, 7]. Lastly, the condition of chronic hypoxia can lead to an inhibition of both innate and adaptive antitumor immune responses [8–10].

### Physical and biological characteristics of carbon ion radiotherapy in cancer treatment

Charged-particle therapy, such as proton radiotherapy (PRT), helium therapy, and carbon ion radiotherapy (CIRT), possesses distinct physical characteristics that make it superior to photon therapy. These characteristics include a sharp lateral penumbra; minimal energy deposition within the beam’s entry path prior to the Bragg peak, which is defined by its steep dose deposition; and a sharp dose-deposition fall-off after the Bragg peak. Thus, charged-particle beams exhibit both a precise and a finite range with respect to their dose delivery capability. The depth of the Bragg peak can be altered by altering the particle beam’s energy. These properties enable the sparing of surrounding normal tissues, which is crucial when irradiating the brain and its critical structures. Several reports have shown improved dose distributions using particle therapy for primary or recurrent glioma, with a suggested improved efficacy and acceptable toxicity profiles [11–13].

In addition to its superior dosimetric properties, a heavy ion such as the carbon ion is a high linear energy transfer (LET) modality. Furthermore, the relative biological effectiveness (RBE) of CIRT is substantially higher than that of photon- and proton-based irradiation. The value of RBE is suggested to be 3–5 for carbon ion. The actual calculated value is dependent on both the tissue type and the biological endpoint of RBE assessment. High-LET irradiation inflicts more damage via direct DNA double-strand breaks, which are more difficult to repair [14]. Improved efficacy could be expected after the delivery of high-LET radiation, such as CIRT, especially in the treatment of photon-resistant cancer cells that have been selected for their ability to more efficiently repair single-strand DNA breaks. Furthermore, several in vitro studies have shown greater tumor-killing efficiency in GBM and, more importantly, in glioma stem cell lines by CIRT when compared to photon radiotherapy or PRT [15–18]. Per convention with CIRT (and other particle-based modalities), the differences in RBE and LET between CIRT and photon radiotherapy is taken into account, and the CIRT doses are reported in terms of gray equivalents (GyE), which refer to the biological effective doses (BEDs) of photons.

The efficacy of CIRT is also much less hindered by hypoxic conditions as compared with photon or proton radiotherapy, which predominate in GBM tumors [19–21]. This, in turn, leads to a greater tumor cell killing efficiency, especially in the early course of treatment, when the greatest number of residual tumor cells are present within the unresected tissue, and along with postoperative tissue reaction, the greatest extent of tumor hypoxia is encountered during the radiotherapy course. Not only does the more densely-ionizing CIRT lead to a greater proportion of tumor cells being killed, the mechanism of cell death is significantly different when compared with conventional, low-LET irradiation, which includes greater ability of inducing the ceramide pathway and increasing complex double-stranded DNA damage which leads to greater autophagy, apoptosis, and mitotic cell death [22–25]. Not surprisingly, this enhanced mode of cellular destruction releases more tumor-specific antigens, is more immunogenic, and fosters a stronger local/direct and abscopal antitumor immune response [26–28].

### Clinical experience of CIRT—safety and efficacy

Results from retrospective and prospective studies have shown improved outcomes after CIRT for several malignancies, including chordoma/chondrosarcoma of the skull base [29–31], melanoma [32], and adenoid cystic carcinoma of the head and neck region. These results also demonstrated the safety of CIRT in protecting the critical organ-at-risks (OARs), such as the optical nerve/chiasm, brain, brainstem, and spinal cord. At the Heidelberg Ion-Beam Therapy Center (HIT) and the National Institute of Radiological Sciences (NIRS) in Japan, CIRT is currently the routine treatment for patients with these mentioned conditions.

### Induction CIRT boost rationale

The novel approach of administering the CIRT boost prior to the initiation of standard, low-LET-based chemoradiotherapy has the following multiple theoretical advantages:

1. Overcoming hypoxia. In the postoperative setting, the number of residual tumor cells and level of hypoxia are at their greatest extent, whereas CIRT can overcome such hypoxic conditions [29];
2. Targeting glioma stem cells earlier in the treatment course. This enables the radiation-naïve stem cells to receive large-fraction doses of the more-effective high-LET irradiation before they can develop radio-resistance to smaller factions of low-LET radiotherapy;
3. Altering the cell death mechanisms/immunogenicity. CIRT can shift the pro-/anti-tumor immunologic balance towards anti-tumor immunity at the beginning of radiotherapy, as opposed to being given after the immunosuppressive TMZ and conventional fractions of low-LET radiotherapy have been delivered;
4. Treatment compliance. Patients are more likely to receive the entire prescribed CIRT dose if it is given first. Fatigue and toxicity of the 30-fraction PRT course could potentially dissuade continued treatment;
5. CIRT responses. Assessing the CIRT-specific responses (imaging and immunologic assays) is less confounded by standard PRT. The biological effect of CIRT differs from that of low-LET PRT and the administration of CIRT boost prior to standard chemoradiotherapy would reduce the interference of other factors to detecting CIRT-specific responses.

## Methods and design

### Study design

The purpose of this trial is to first determine, in a Phase I dose escalation study, the maximal safe induction CIRT boost to the residual tumor in malignant glioma patients [GBM or anaplastic astrocytoma (AA)] that can be given to patients in addition to adjuvant radiotherapy at the standard dose (i.e., 60 GyE in 30 fractions). The rationale for this approach is to provide a different form of radiotherapy; one with a higher LET, having a different mode of tumor-killing, and possessing a greater overall tumor-killing ability due to its weaker dependence on oxygenation for efficacy, to the site of disease immediately after surgery when the number of tumor cells and extent of hypoxia are at their greatest. We anticipate that these factors will result in a shift from the usual pro-tumor immunosuppression, fostered by tumor microenvironment, into one that promotes a greater inflammatory and anti-tumor immunity environment.

In the second stage of this trial, the Phase III, the following hypothesis will be tested: When induction CIRT boost with the maximal safe dose (determined in Phase I) is given prior to standard PRT, whether this strategy will result in a prolonged OS of GBM patients without provoking additional toxicity as compared with standard PRT.

A secondary aim of this study is to determine if there are immunologic response profile differences between those receiving CIRT boost and those who do not, such that additional immunotherapeutic agents can be administered in future trials to synergize and augment the effects of the CIRT boost.

### Ethics, informed consent, and safety

All clinical trials of the SPHIC, including the present trial, are required to adhere to the policies set forth by the Institutional Academic Committee and the Institutional Review Board (IRB). IRB approval of the current trial was obtained. The following sections were adapted and summarized from the institutional policies on human studies to which the present trial will adhere.

The IRB of the Shanghai Proton and Heavy Ion Center (SPHIC) independently monitor the recruitment, the reporting of adverse events, and the data quality for all clinical trials. Data and interim results for this trial will be reviewed semi-annually according to the trial protocol. The IRB will provide the principal investigator with requirements and recommendations on the modification of the trial, which may or may not include termination of the trial.

The study will be conducted according to the Chinese guidelines for good clinical practice (GCP) and the principles of the Declaration of Helsinki (2008 version, adopted at the 59th World Medical Association General Assembly, Seoul, Korea, October 2008).

### Trial design

#### Phase I

In this initial portion of the study, the maximal tolerable dose (MTD) of the induction CIRT boost will be determined. Anaplastic astrocytoma (AA) will be allowed to be included in the Phase I study, as it will expedite the accrual time and considering that this tumor is treated identically as to GBM at our institution. The starting boost dose will be 9 GyE in 3 fractions for the first three patients enrolled. In the absence of any severe (defined as ≥ grade 3) acute toxicities [dose-limiting toxicity (DLT)] assessed using the Common Terminology Criteria for Adverse Events (CTCAE) v. 4.03, the next 3 patients will receive 12 GyE in 3 fractions (i.e., 4 GyE × 3 fractions). Further dose escalation will be continued to 15 GyE then the maximal 18 GyE in 3 fractions (i.e., 5 GyE then 6 GyE × 3 fractions). The MTD will then be used in the experimental arm in Phase III of this trial. If there is one case of ≥ grade 3 acute toxicity within a dosing group, 3 more patients will be added to this group. If 2 or more cases of ≥ grade 3 acute toxicities are observed, the boost dose used in the preceding lower-dose group will be chosen. If 9 GyE proves to be too toxic, a CIRT boost of 6 GyE in 2 fractions will be incorporated into the experimental arm. All Phase I patients, after receiving induction boost irradiation, will go on to receive 60 GyE in 30 fractions of PRT with concurrent daily TMZ at 75 mg/m^2^. Table 1 summarizes the Phase I design.

**Table 1:** Phase I design of dose escalation of induction carbon ion boost for treating GBM and AA

Remarks	Boost set	Induction carbon ion Boost			Cumulative BED^*^ (GyE)		EQD2^*^ (GyE)	
		Dose/Fx (GyE)	No. of Fx	Total dose (GyE)	Tumor (α/β = 10)	Brain (α/β = 3)	Tumor (α/β = 10)	Brain (α/β = 3)
Phase I starting dose	1	3	2	6	79.8	112.0	66.5	67.2
	2	3	3	9	83.7	118.0	69.8	70.8
	3	4	3	12	88.8	128.0	74.0	76.8
	4	5	3	15	94.5	140.0	78.8	84.0
	5	6	3	18	100.8	154.0	84.0	92.4
PRT regimen	N/A	0	0	0	72.0	100.0	60.0	60.0

*GBM* glioblastoma, *AA* anaplastic astrocytoma, *BED* biological effective dose, *EQD2* equivalent dose for a 2 GyE/fraction treatment, *α/β* alpha/beta ratio, *Fx* fraction, *GyE* gray-equivalents

* The dose of proton radiotherapy (60 GyE in 30 fractions) is included in the calculation of cumulative BED and EQD2

#### Phase III

##### Experimental arm

A total of 122 patients will be enrolled into this arm and undergo an induction CIRT boost to the residual gross tumor volume. The dose of the induction boost will be determined in Phase I of the trial. One week after the first fraction of the induction boost, a standard PRT (60 GyE in 30 fractions) with concurrent daily oral administration of TMZ at 75 mg/m^2^ will commence.

##### Control arm

A total of 121 patients will be enrolled into this arm and undergo standard PRT with concurrent daily oral administration of TMZ.

##### Serologic immune markers

**Fig. 1:**
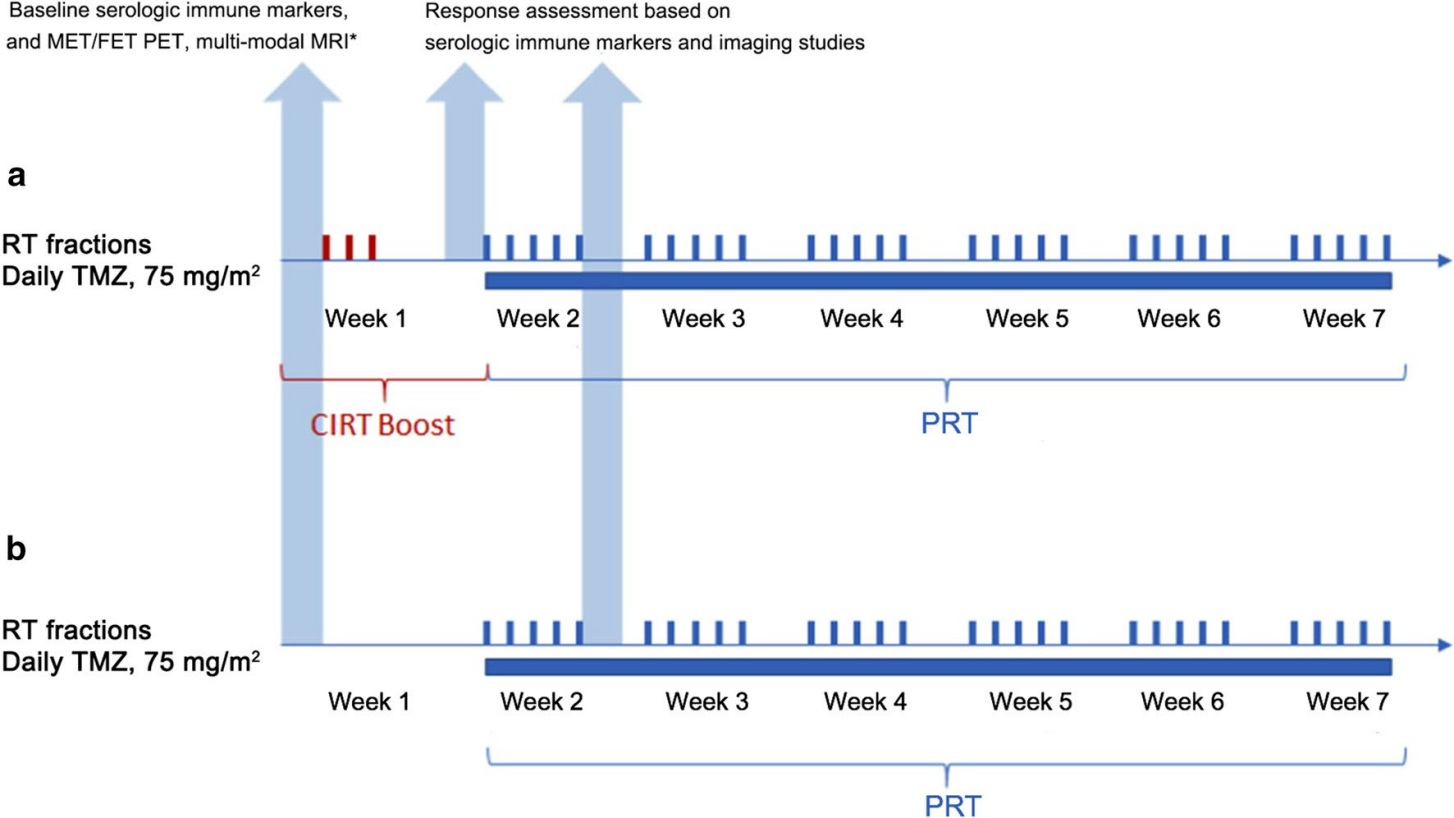
Illustration of the overall schema of the Phase III of the current trial. In the Phase III, the GBM patients will be randomized to receive either **a** a CIRT boost followed by standard PRT with concurrent TMZ (experimental arm) or **b** standard PRT with concurrent TMZ (control arm). Each patient will undergo an assessment of their tumor response based on imaging and immunologic serum studies. *Multi-modal MRI includes MRS, BOLD, DWI, DTI, PWI, and MRI. *GBM* glioblastoma, *CIRT* carbon ion radiotherapy, *PRT* proton radiotherapy, *TMZ* temozolomide, *RT* radiotherapy, *MET/FET PET* C-methionine positron/18F-fluoro-ethyl-tyrosine positron emission tomography, *MRI* magnetic resonance imaging, *MRS* magnetic resonance spectroscopy, *BOLD* blood oxygenation level-dependent imaging, *DWI* diffusion-weighted imaging, *PWI* perfusion-weighted imaging

To ascertain the specific immune response profile to the induction CIRT boost, all patients enrolled into the Phase III study will have their blood samples investigated before irradiation to establish the baseline profile of serologic immune markers (detailed in Table 2 and between the fifth and sixth fractions of PRT to determine the changing profile. Those who receive the induction CIRT boost will have an additional serologic marker detection between the last fraction of the boost and the first proton fraction. The overall Phase III schema is illustrated in Fig. 1.

**Table 2:** Serologic immune markers of GBM

Marker	Function/correlative findings	Assay
Pro-inflammatory/anti-tumor		
IL-1β	Stimulates cytotoxicity; serum levels decrease during low-LET radiotherapy [55]	Serum ELISA
IL-6	Pro-inflammatory cytokine, defective expression from lymphocytes in patients with glioma [56]	Serum ELISA
TNF-α	Promotes cell-directed cytotoxicity; reduced levels are associated with GBM [57]	Serum ELISA
CD3+ lymphocytes	Direct immune-mediated cytotoxicity; increased levels are associated with prolonged survival [58]	Flow cytometry
CD8+ lymphocytes	Direct immune-mediated cytotoxicity, increased levels are associated with prolonged survival [58]	Flow cytometry
Anti-inflammatory/pro-tumor		
IL-4	Induces immunosuppression and tumor tolerance; high levels are associated with GBM [57]	Serum ELISA
IL-10	Induces immunosuppression and tumor tolerance; high levels are associated with GBM [57]	Serum ELISA
TGF-β	Promotes proliferation and immune escape of GBM [59]	Serum ELISA
Regulatory T cells	Secrete IL-10 and TGF-β, suppress CD8-dependent tumor-specific cytotoxicity, and elevated in peripheral blood of GBM patients [60]	Flow cytometry

*GBM* glioblastoma, *IL* interleukin, *TNF*-*α* tumor necrosis factor-α, *TGF*-*β* transforming growth factor-β, *LET* linear energy transfer, *ELISA* enzyme-linked immunosorbent assay

### Study objectives

#### Phase I

The primary objective is to determine the maximal safe induction CIRT boost dose.

#### Phase III

The primary objective is to detect an improvement in OS in those patients who received an induction CIRT boost with no additional toxicity. The secondary objectives are to determine the response rates, progression-free survival (PFS), and tumor response (based upon assessment with C-methionine/fluoro-ethyl-tyrosine positron emission tomography [MET/FET PET] or magnetic resonance imaging [MRI] and detection of serologic immune markers).

### Patient selection

#### Inclusion criteria

Patients who meet all of the following criteria will be considered for recruitment into this trial:

- Histologically confirmed, unifocal, supra-tentorial primary AA or GBM;
- Residual, clinically measurable tumor up to 5 cm in the largest dimension assessed by postoperative MET/FET PET, MR spectroscopy (MRS), or MRI;
- Able to determine the *MGMT* promoter methylation status;
- Indication for adjuvant radiotherapy with concurrent TMZ administration;
- Age ≥ 18 years;
- Karnofsky performance score ≥ 60;
- Ability to understand the purpose and content of the clinical trial;
- Written informed consent with required signature prior to enrollment and initiation of the treatment.

#### Exclusion criteria

Patients who present with any of the following criteria will not be included in this trial:

- Patient’s refusal to follow the trial protocols;
- Severe pulmonary hypertension, cardiovascular disease, peripheral vascular disease, severe chronic heart disease, and other complications that may interfere with radiotherapy;
- Previous radiotherapy to the brain;
- Previous malignancy requiring cytotoxic therapy within 5 years prior to enrollment;
- No residual, clinically measurable disease observed on postoperative MET/FET PET, MRS or MRI;
- A time interval > 8 weeks between surgery and the initiation of radiotherapy;
- Not yet recovered from toxicities of prior treatments;
- Pregnant or lactating women;
- Participation in another clinical study or in an observation phase of a competing trial.

### Treatment assignment

**Fig. 2:**
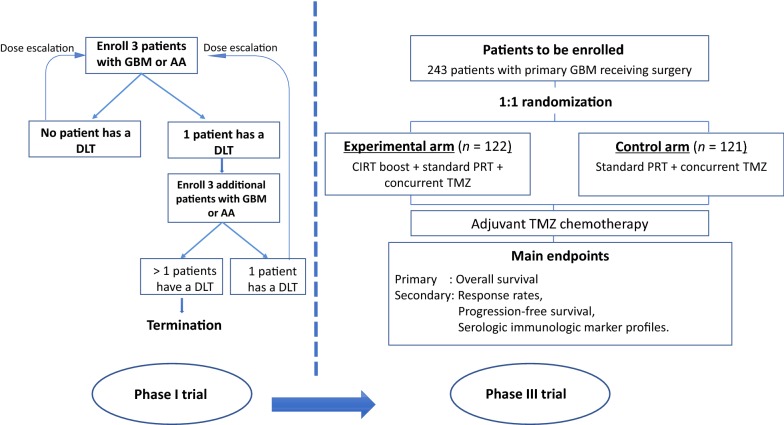
The flow chart of the current Phase I/III trial. In the Phase I, the maximal tolerable dose (MTD) of the induction CIRT boost will be determined. The MTD will then be used in the experimental t arm in the Phase III of this trial. The Phase III aims to determine the overall survival, progression-free survival, and tumor response. *GBM* glioblastoma, *AA* anaplastic astrocytoma, *DLT* dose-limiting toxicity, *CIRT* carbon ion radiotherapy, *PRT* proton radiotherapy, *TMZ* temozolomide

The flow chart of this Phase I/III trial is illustrated in Fig. 2. Patients enrolled into Phase I of this trial will be assigned to groups of 3, and each group will receive a progressively increasing dose of induction CIRT boost until the safe dose is determined or the maximal dose of 18 GyE in 3 fractions is reached without ≥ grade 3 toxicities. Patients who later opt out of the study will be replaced by newly enrolled ones. Patients enrolling into the Phase III of this trial will be randomly assigned to either the experimental or control arm (1:1 randomization), as described above. Patients withdrawn from this phase will retain their randomization number, and new patients will be issued with a new randomization number.

### TMZ dosing and schedule

Conventional oral chemotherapy with TMZ (75 mg/m^2^) will commence on the first day of PRT in both arms. TMZ will not be administered during the CIRT boost in the experimental arm so as not to suppress the desired shift towards an anti-tumor immune response and the effect against glioma cells that have infiltrated the brain parenchyma at a distance from the primary tumor site.

### Radiotherapy planning

Target volume delineation: Numerous studies have indicated that the use of MET/FET PET, MRS in conjunction with MRI was superior to MRI alone in determining the extent of malignant involvement [33–36]. Therefore, tumor areas presented on the MET/FET PET, MRS as well as the traditional contrast enhancement and fluid-attenuated inversion recovery (FLAIR) abnormality indicative of residual non-enhancing tumor (edematous FLAIR signal that does not resolve after surgical decompression) will be used to determine the gross tumor volume (GTV).

The clinical target volume (CTV) receiving 60 GyE irradiation (CTV60) will be delineated with a 0.5-cm margin expansion from the GTV and will be the principle planning target volume of PRT. During PRT, a secondary CTV of 50 GyE in total will be given. The CTV50 will be delineated with a 1.5-cm expansion from the GTV. The volumes, doses, and delineations are summarized in Table 3.

**Table 3:** Target Volume delineations and radiotherapy planning of GBM and AA

Radiotherapy	Target volume	Delineation	Dose/fractionation	Minimal dose coverage
CIRT boost	GTV	MET/FET PET or MRS abnormality, contrast enhancement, FLAIR abnormality representing residual tumor	3.00-6.00 GyE × 3 Fx	95%
PRT	GTV	MET/FET PET or MRS abnormality, contrast enhancement, FLAIR abnormality representing residual tumor	N/A*	N/A*
	CTV60	GTV + 0.5 cm margin	2.00 GyE × 30 Fx	95%
	CTV50	GTV + 1.5 cm margin	1.67 GyE × 30 Fx	95%

*GBM*: glioblastoma, *AA* anaplastic astrocytoma, *CIRT* carbon ion radiotherapy, *GTV* gross tumor volume, *MET/FET PET* C-methionine/18F-fluoro-ethyl-tyrosine positron emission tomography, *MRS* magnetic resonance spectroscopy, *FLAIR* fluid-attenuated inversion recovery, *N/A* not applied, *GyE* gray-equivalents, *Fx* fraction, *PRT* proton radiotherapy, *CTV60/50* clinical target volume receiving 60/50 GyE irradiation

* N/A: Since the GTV of PRT is not a target volume to give prescription dose, so “N/A” is marked in these cells

CIRT and PRT planning: Radiotherapy will be planned using the Syngo treatment planning system (Siemens, Erlangen, Germany) and delivered using the IONTRIS intensity-modulated raster scan system (Siemens, Erlangen, Germany) as previously described [37]. Briefly, CIRT and PRT will be performed once a day, 5 days a week. The patient’s position will be verified using a craniofacial X-ray before radiotherapy, and set-up deviations of more than 2 mm will be corrected. CIRT is planned with RBE calculated using the local effect model (LEM). The RBE values of CIRT at 3, 4, 5, and 6 GyE will be 3.3, 2.9, 2.6, and 2.4, respectively.

### Clinical outcome assessments

#### Overall survival

The duration of OS will be calculated as the time between the pathological confirmation of GBM and the date of death from any cause. Patients who do not succumb to their disease or who are not lost to follow-up will be censored at the date of the last follow-up.

#### Progression-free survival

PFS will be defined as the time between the first day of treatment and the date of disease recurrence confirmed by imaging (either on routine follow-up or symptom-directed imaging studies). Patients who do not succumb or who are not lost to follow-up and present with no evidence of disease progression will be censored at the date of the last follow-up.

#### Toxicity rates

The CTCAE v. 4.03, or the most recent update, will be used to assess all toxicity and adverse events observed during and after the completion of treatments [38]. Patients will be evaluated weekly by complete history taking, physical examination, and blood chemistry tests to determine the safety and toxicity of all treatments in this clinical trial. These evaluations will be conducted at each on-treatment appointment and at each clinical follow-up visit.

Efforts will be made to use bevacizumab as a first-line treatment for radiation-induced tumor/cerebral edema, based on its efficacy and minimal immunosuppressive effects [39–42]. For refractory cases or those in which bevacizumab is not available, dexamethasone will be used per routine protocols and noted in conjunction with serologic immune marker studies.

### Treatment response assessments

#### Imaging

To assess the initial responses to the CIRT boost, MET/FET PET or MRS will be performed at 2 weeks after the initiation of CIRT in the experimental arm. To assess the overall treatment response, MET/FET PET or MRS will be performed at 4 weeks after the completion of PRT in both arms. The target lesion will be evaluated using the response assessment in neuro-oncology (RANO) criteria [43] with interpretation modifications [44, 45] and is summarized below:

- Complete response (CR): Complete disappearance of all tumors on consecutive computed tomography (CT) or MRI scans sustained for at least 1 month, without the use of steroids.
- Partial response (PR): An observed ≥ 50% decrease in the area of contrast enhancement on consecutive CT or MRI sustained for at least 1 month. Doses of steroids must be stable or decreased, and the patient must be neurologically stable.
- Progressive disease (PD): An observed ≥ 25% increase in the area of contrast enhancement or any new tumor on CT or MRI.
- Stable disease (SD): All other situations.

#### Serologic immune markers

Because it is anticipated that the immune response towards GBM treated with CIRT will be distinct from the response towards tumors treated with standard, low-LET radiotherapy, peripheral blood levels of key immunologic markers will be assayed and compared with baseline levels. Several studies have reported that aberrant levels of cytokines and subtypes of circulating T cells can be detected in the blood samples of glioma patients and can serve as markers of tumor biology/immune state, virulence, and progression [46–49]. The markers to be assayed play critical roles in either the pro-inflammatory/anti-tumor or anti-inflammatory/pro-tumor immunity and are summarized in Table 2. The dual purpose of this data collection will be to both characterize the immune responses towards GBM treated with CIRT and to inform future immunotherapy trials which use this radiotherapeutic modality.

### *MGMT* promoter methylation status

When possible, each enrolled patient will have their tumor tissue specimens investigated to assay the presence or absence of *MGMT* promoter methylation using methylation-specific polymerase chain reaction (PCR) as previously described [50]. *MGMT* promoter methylation occurs in approximately 50% of all GBM patients. It is associated with the biologically aggressive secondary and pro-neural subtypes and, not surprisingly, represents a favorable prognostic and predictive factor for an improved response to both irradiation and TMZ [51]. The presence of this molecular biomarker will be subjected to correlative, subset outcome and response analyses.

### Treatment after tumor progression

After completion of chemoradiotherapy, further treatment, including surgical resection, a second course of irradiation, systemic chemotherapy, or targeted therapy may be clinically necessary in case of disease recurrence or progression. These treatment decisions will be made at the discretion of the treating physician and/or team.

### Follow-up

After completion of treatment, all patients will be required to be followed-up regularly, indefinitely or until death, according to our institutional follow-up protocol. The first and second follow-up visits will be scheduled at 1 and 3 months after the completion of radiotherapy. Unless otherwise clinically necessary, follow-up sessions will then be scheduled every 3 months in the first 3 years, every 6 months in the following 2 years, and annually thereafter. Each follow-up will include a complete history taking and physical examination, MRI of the brain, and blood chemistry tests (including complete blood counts, serum electrolyte levels, and liver/renal function test).

### Statistical methods

#### Treatment effect size

The Phase III of this trial will be designed to detect a prolongation of 8 months in survival. The rationale behind this assumption is as follows: CIRT is characterized by a higher RBE as compared with conventional photon radiotherapy or PRT. Preclinical experiments on glioblastoma cell lines have shown that the RBE of CIRT lies between 3 and 5, depending on the respective endpoint or cell line [52]. Further, the timing of the CIRT boost (given prior to standard PRT) is expected to overcome the tumor environmental limitations (hypoxia, radio-naïve cells, cell number, etc.) with greater cell-killing effect than conventional PRT alone. Therefore, the effect of CIRT on primary glioblastoma is expected to be substantially higher than that of conventional PRT alone.

#### Type I error rate and power

Time to death for any reason will be compared between the two arms in Phase III of this trial, using a two-tailed log-rank test at an overall type I error rate of 5%. The desired power is 80%. Sample size calculation is based on the unstratified log-rank test.

#### Sample size calculation

For calculating the OS, assuming a median OS of 16 months in the control arm and 24 months in the experimental arm (corresponding to a hazard ratio of 0.67 or a reduction in the risk of death by 33%), 192 events are required to achieve an 80% power of the log-rank test at a two-sided overall α level of 5%.

#### Anticipated duration of trial

The sample size for this trial is calculated based on the assumption of a 36-month recruitment period (with an average recruitment of 7 patients per month) and a minimal follow-up of approximately 24 months for the last patient enrolled. To observe the required number of events in the timeframe defined above, a total of 243 patients (122 in the experimental arm and 121 in the control arm) will be required. A dropout rate of 5% (12 patients) due to lost to follow-up or treatment discontinuation has been considered in the above-mentioned estimations.

#### Analytical techniques

##### Outcome analyses

The difference in duration of survival between the two arms will be tested with a two-sided, stratified log-rank test at the 5% α level. Kaplan–Meier curves will be displayed, and median survival estimates and confidence limits will be calculated. The Cox regression analyses, adjusted and unadjusted, for stratification factors will be performed in an exploratory manner. PFS will be analyzed analogously to the method for OS, but of explorative nature.

A Lan-DeMets α spending function with an O’Brien-Fleming boundary will be used at the interim analysis to maintain an overall α of 0.05. The interim analysis will be performed after 96 OS events with an α of 0.003; the primary OS analysis will be performed after 192 events with an α of 0.049.

The rates of adverse events will be summarized by the type and severity. A Fisher’s exact test will be used to compare the observed adverse events between the two arms.

The primary efficacy analysis will be performed in the intention-to-treat (ITT) population, which is defined as the population of all randomized patients, analyzed in the arm to which they have been assigned. The OS and PFS of the ITT population will be calculated. The toxicity analysis will be performed on all patients who have received at least one dose of irradiation.

##### Biomarker analyses

Statistical analyses will also be performed to identify the associations between molecular marker profile and OS, PFS, and response rates. In univariate analysis, the log-rank test will be used to test for differences in OS and PFS between the different subgroups defined by the *MGMT* promoter methylation status and serologic marker levels. Multivariate analyses will be performed using the Cox proportional hazard model to determine if the molecular markers are independent prognostic factors and possibly predictive factors for treatment effect.

#### Data handling, storage, and archiving

The Chinese GCP regulation requires that all clinical trial documents must be kept for at least 5 years after completion or termination of the trial. The Shanghai Municipal Health Commission requires that all patient medical charts, including all imaging records, to be maintained for at least 7 years. The Research Unit and the Medical Record Unit of the Department of Medical Affairs of the SPHIC will be responsible for archiving the research documents and medical charts, respectively.

#### Interim analysis

To allow for early stoppage of the trial, in case of an observed overwhelming treatment effect, a group-sequential design will be applied with one interim analysis that is to be performed when 50% of the expected number of events under the null-hypothesis have occurred. Under the assumptions made in sample size calculation, the interim analysis will be performed after an occurrence of 92 events, which should happen approximately 26 months after the initiation of the recruitment. The stopping rule is specified according to O’Brien and Fleming [53].

No formal boundary for stopping for futility is specified. However, if the results of the interim analysis suggest that the objectives of the study cannot be reached with a feasible number of patients or that the benefit/risk ratio for the study has worsened markedly, the study may be stopped by the decision of the principle investigator. As in this case, the null-hypothesis would not be rejected, no type I error would be committed, and therefore the type I error rate of the study would still be controlled at 5%.

## Discussion

The results of traditional tri-modality treatment of GBM (maximal safe resection, low-LET photon irradiation with concurrent TMZ) remain discouraging. The recent addition of the alkylating agent, TMZ, prolonged the median survival from 12 months to 15 months [1]. It was the first time that a change in treatment has lead to a statistically significant improvement in OS since the past several decades, but the efficacy is still not satisfactory. However, there have been no new equally substantial breakthroughs in the past 12 years since the release of the results from that study, despite exhaustive efforts to build upon its progress have been tried. Clearly, a more radical and “outside-of-the-box” strategy is needed.

Our proposed clinical trial is novel and may represent a first step in a new direction for several reasons. First, it uses CIRT, which has a multitude of physical properties that set itself apart from both the traditional photon-based irradiation and even the more widely-used, particle-based PRT. Like PRT, CIRT is characterized by a low dose deposition along the entry channel of the beam, with a steep dose deposition in the region of the Bragg peak, which by modulation of the beam energy and tissue compensation, can be focused over CTV. The rapid dose deposition fall-off, after the Bragg peak, allows for dose-sparing of normal tissue and non-targeted structures, significantly reducing toxicity. Unlike photon radiotherapy or PRT, CIRT poses both high-LET and RBE properties, which translate into more efficient tumor cell killing (even in conditions of hypoxia and against low-LET resistant tumors) and via distinct mechanisms, which have been shown to be anti-tumor immunogenic and to induce abscopal effects.

Second, and perhaps the most important reason, is that CIRT is used as a boost prior to the initiation of the standard treatment course, which consists of low-LET PRT with concurrent TMZ. The special timing of the boost will take full advantage of the unique characteristics of CIRT. From a tumor cell population perspective, the postoperative setting, before the initiation of adjuvant radiotherapy, represents the point in time when the number of residual cells is the largest and when the hypoxia levels are at their greatest. Therefore, it can be theorized that this is when the more efficient tumor cell-killing CIRT should be used since its biological impact will not be hindered by hypoxia. Further, large fractions of this high-LET irradiation, when applied to radio-naïve cells (including glioma stem cells), will result in a more robust initial cell-killing effect. In the absence of immunosuppressive effects of TMZ, we anticipate that the immunogenic response will shift the balance from a pro-tumor immunity (common in GBM) to an anti-tumor immunity and will proceed unperturbed.

The abscopal effect, observed to be induced when CIRT is applied to other tumors may have importance in GBM. The diffuse, infiltrative nature of glioma cells create a scenario where the tumor and tumor-initiating cells are disseminated throughout the brain parenchyma, outside of radiotherapeutic target volumes, and can evade lethal doses of irradiation. Further, they can migrate towards the primary tumor site and initiate tumor recurrence [54]. It is tempting to speculate that the immunogenic nature of CIRT could create an abscopal effect within the brain’s distinct immune system and that these malignant cells could be more efficiently cleared by anti-tumor immunity. We anticipate that this phenomenon will contribute towards improved outcomes for the patients enrolled in the experimental arm of the Phase III of this trial.

The Phase I of this trial allows for the inclusion of AA patients. This is permissible for several reasons. Patients with AA are treated in an identical fashion at our institution, and the purpose of the Phase is to determine the maximal safe dose of CIRT boost, which is not dependent upon the histology of the tumor. However, AA patients are excluded from Phase III of this trial, as their expected median OS ranges from 2 to 3 years, which are significantly longer than that of GBM patients, and their inclusion within the statistically-weighted study design could interfere with the final study results.

In summary, the CIRT boost delivered prior to standard chemoradiotherapy is a novel therapeutic approach for GBM. It uses the relatively new modality of radiotherapy. In addition, the timing of its delivery seeks to maximize the physical, biological, and immunologic advantages that CIRT has over the more traditional, low-LET radiotherapies.

## Abbreviations

AA: anaplastic astrocytoma
BED: biological effective dose
CIRT: carbon ion radiotherapy
CTV: clinical target volume
CR: complete response
DLT: dose-limiting toxicity
ELISA: enzyme-linked immune-sorbent assay
EQD2: equivalent dose for a 2 Gy/fraction treatment
FLAIR: fluid-attenuated inversion recovery
GBM: glioblastoma
GCP: good clinical practice
GTV: gross-tumor volume
GyE: gray equivalents
HIT: Heidelberg Ion-Beam Therapy Center
IL: interleukin
IRB: Institutional Review Board
ITT: intention to treat
LET: linear-energy-transfer
MET/FET PET: C-Methionine/18F-fluoro-ethyl-tyrosine positron emission tomography
*MGMT*: O-6-methylguanine-DNA methyltransferase
MTD: maximal tolerable dose
MRS: magnetic resonance spectroscopy
NIRS: National Institute of Radiological Sciences
OAR: organ-at-risk
OS: overall survival
PCR: polymerase chain reaction
PRT: proton radiotherapy
PR: partial response
RBE: relative biological effectiveness
SD: stable disease
SPHIC: Shanghai Proton and Heavy Ion Center
TGF-β: transforming growth factor beta
TNF-α: tumor necrosis factor α
